# Editorial: Antibody Repertoire and Graft Outcome following Solid Organ Transplantation

**DOI:** 10.3389/fimmu.2017.00648

**Published:** 2017-06-09

**Authors:** Narinder K. Mehra, Ajay Kumar Baranwal, Brian D. Tait

**Affiliations:** ^1^All India Institute of Medical Sciences, New Delhi, India; ^2^Australian Red Cross Blood Service Australia, Melbourne, VIC, Australia

**Keywords:** antibody, graft, organ transplantation, anti-HLA antibodies, sMICA

**The Editorial on the Research Topic**

**Antibody Repertoire and Graft Outcome following Solid Organ Transplantation**

In recent years, there has been vast improvement in the survival of solid organ grafts. Improved immunosuppression has resulted in efficient control of cell-mediated immune responses and has also mitigated the effects of HLA mismatching between the recipient and the grafted organ. Antibody-mediated rejection (AMR) however still remains a clinical challenge and the identification of donor-specific HLA antibodies pretransplant are a mandatory requirement for successful grafting. Accurate and sensitive HLA antibody identification has been made possible by the introduction of solid phase assays such as the Luminex bead based platform for antibody testing.

Despite this advancement in detecting and understanding the role of HLA antibodies in rejection, many questions still require answers in order to maximize the outcome in solid organ transplantation. Many of these aspects are dealt with efficiently in this excellent series of papers addressing the role of antibodies in solid organ rejection.

This collection of reviews, some including new findings, by world leaders in their particular subspecialty, represents a compendium of the most recent and exciting developments of special interest to those who are involved in understanding the role of antibodies in organ rejection.

The technical aspects of antibody detection have been addressed in detail by Tait (Melbourne). While solid phase and, in particular, the Luminex bead assays have revolutionized the way antibody screening is now conducted, they have produced their own particular challenge. The failure to discriminate complement from non-complement fixing antibodies by solid phase assays poses a problem with respect to the significance of a positive result, which has been partially addressed by modification of the Luminex assay to measure complement fixation.

Data from clinical studies suggest that non-complement fixing antibodies may compromise graft outcome, albeit not to the same extent as complement-fixing HLA antibodies. The presence of antibodies directed at denatured HLA and detected by the Luminex platform, although not clinically important can also complicate the analysis of antibody specificity determination. Issues such as the importance of the mean fluorescence index on graft outcome and the value of the virtual cross match are also discussed in this review.

The functional aspects and clinical impact of alloantibodies and autoantibodies are discussed in several excellent reviews.

Despite the best efforts of avoiding pretransplant HLA antibodies, there is no way of predicting which patients will develop *de novo* antibodies. Mangiola and co-workers (Pittsburgh) discuss the role of both pretransplant and *de novo* HLA antibodies in pediatric and adult heart transplant recipients. They stress that the treatment for early and late rejection are different, which raises the important question of the optimal protocol for posttransplant screening in order to maximize timely detection of antibodies prior to organ damage.

In addition to HLA antibodies, the role of non-HLA antibodies is discussed by several author groups. Matsuda and Sarwal (San Francisco) emphasize the point that antibody-mediated chronic rejection remains the biggest unresolved issue. Chronic AMR has been shown in cases involving HLA identical recipients and donors suggesting a role for non-HLA antibodies. This review is a “tour de force” covering mechanisms underlying antigen recognition, the role of both HLA and non-HLA antibodies in rejection and immunosuppressive approaches.

Nayak et al. (Phoenix) studied both auto- and alloantibodies in lung and pancreatic islet cell transplantation, which are more susceptible to rejection by a combination of antibodies than other forms of transplants. Islet cell transplant patients are often exposed to multiple islet cell infusions and as a result are exposed to many HLA class 1 and 2 mismatches. In addition to HLA immunity, there is evidence that autoantibodies to GAD65 and autoreactive T cells participate in the rejection process. It appears that once tolerance is broken to the self-antigens, then immunosuppression becomes ineffective. In lung transplantation, HLA antibodies are associated with obstructive airway disease while the appearance of MICA antibodies after HLA donor-specific immunization is associated with bronchiolitis obliterans syndrome.

Zhang and Reinsmoen (Los Angeles) have submitted a comprehensive review on the role of non-HLA antibodies in kidney and heart transplant rejection. Antibody specificities discussed include those to myosin, vimentin, Kα1 tubulin, collagen, and angiotensin II type 1 receptor. There appears to be synergy between HLA and non-HLA antibodies. In some cases, development of HLA antibodies precedes the development of antibodies to non-HLA antigens. It is suggested that the damage inflicted on the graft by HLA antibodies exposes cryptic antigens on non-HLA molecules that result in antibody formation by the recipient. There is also evidence that the presence of non-HLA antibodies may predispose the recipient to the formation of HLA antibodies. Given this close relationship between HLA and non-HLA antibodies, the authors stress the point that it is imperative that both types of antibodies be measured in transplant recipients.

Baranwal and Mehra (New Delhi) discuss the importance of MICA antibodies on graft outcome in a thorough review of the literature and demonstrate that MICA antibodies are detrimental to the outcome of solid organ transplantation, but soluble MICA appears to have an inverse relationship to rejection. The mechanism behind this observation appears to be the interaction of the soluble MICA with the MICA ligand NKG2D, thus blocking the activation of NK cells. Amino acid position 129 appears to be pivotal in the induction of immunity in renal transplant patients. Recipients who are homozygous for methionine at this position have a higher incidence of rejection than those with valine homozygosity. The mechanism underlying this association is not understood.

Valenzuela et al. (Los Angeles) discuss the impact of IgG subclass on solid organ rejection. The histology and clinical profiles surrounding both HLA and non-HLA donor-specific antibodies is very heterogeneous with limited understanding of the various roles that the IgG antibody subclasses play. The authors describe in detail the various functions that IgG subclasses subserve, the data for which is largely derived from infectious disease studies and human cancers, but make the point that in clinical transplantation there is little known. Analyzing all known data suggests that IgG3 is predominantly associated with complement-mediated rejection while IgG4 is associated with memory, and subclinical and chronic rejection.

One of the complicating issues is that most patients have mixtures of two or more isotypes, which makes analysis of single isotype function difficult. Newer assays are needed that could measure single antibody isotypes, permitting correlations to be established between antibody isotypes and the various features of complex rejection histology.

An intriguing piece of original research by Geneugelijk et al. (Basel) focuses on nature’s allograft, the fetus. They demonstrate that a previous miscarriage prior to a successful second pregnancy produces a lower rate of immunization to paternal HLA than a previous full-term pregnancy. Examination of the number or epitope differences between the mother and fetus revealed the intriguing observation that patients with a previous miscarriage actually had a lower rate of HLA incompatibility. This observation is not readily explained, although the authors have put forward several hypotheses that require further exploration. Suffice to say the mechanism underlying this observation may have some relevance to immunization mechanisms in the clinical allograft situation.

Rajalingam (San Francisco) discusses the role of NK cells in organ rejection. At first sight, this review does not seem central to the theme of the series. However, the link is made by virtue of the fact that the presence of HLA antibody bound to graft endothelium can activate NK cells *via* the antibody-dependent cell-mediated cytotoxicity (ADCC) pathway. Infiltration of grafts with NK cells have been shown in renal, cardiac, liver, and lung transplants indicating a key role for these cells in the rejection process, and NK cell transcripts have been demonstrated in kidney biopsies undergoing AMR.

Morath et al. (Heidelberg) provide an excellent review of ABO incompatible (ABOi) renal transplants with data from the Collaborative Transplant Study (CTS) established by Gerhard Opelz in Heidelberg in 1982. This has been an invaluable resource over four decades comprising 400 participating transplant centers from 42 countries, providing data on kidney, heart, lung, liver, and pancreas transplants.

The concept of a major ABOi renal transplant was an absolute contraindication. However, over the last 25 years, the need to expand living donor options for some patients led investigators into the possibility that with ABO antibody reduction pretransplant, it was possible to transplant across the major ABO barrier. If the ABO titer can be reduced to below 1:32 by plasmapheresis, membrane filtration or immunoadsorption, as measured by the tube method, then although there is a rebound effect posttransplant, it is non-damaging to the graft, a process termed accommodation. Antibody ablation is accompanied by other procedures designed to blunt the rebound response. These include the use of IVIG to modulate the recipient’s immune system and anti-CD20 (rituximab) therapy to reduce the B cell pool. A new approach has been the use of eculizumab, a hybrid monoclonal that is a terminal complement blocker, in this case designed to block the damaging effect of the ABO antibodies bound to the graft endothelium. Results to date however are inconclusive with respect to the efficacy of this approach. A CTS study of 1420 ABOi renal transplants from multiple transplant centers has revealed that while graft survival of ABOi are comparable to ABO compatible grafts, early rejection and the complications of early infection are increased, with one additional patient per 100 dying from this complication in the ABOi.

Interestingly within the ABOi group, the use of anti-CD20 appears to have a significant beneficial effect. Although the use of ABOi transplants has greatly increased the living donor choices for many patients, the authors feel that caution has to be exercised in the use of ABOi due to the complications of infection.

The functional role of B cells has been elegantly addressed by Karahan et al. (Leiden) who stress the point that B cells play other roles in addition to their antibody-producing function. They discuss in detail the mechanisms underlying the role B cells play as cytokine producers and as antigen-producing cells as well as their ability to organize tertiary lymphoid tissue. B cells also invoke T cell immunity and of course regulatory B cells are central to control the immune response. Their pivotal message is that ablation of B cells can be detrimental as well as beneficial. Understanding how the different populations of B cells interact could lead to more rational and targeted immunosuppression.

Wu et al. (Sichuan) discuss the role of IL-21, which is produced by CD4+ T cells and their interaction with both plasma and memory B cells. As with the Karahan et al. paper, the possibility arises that these findings could be used to establish novel immunosuppressive approaches.

Two reviews discuss both historical aspects of HLA matching in solid organ transplantation and the evolution in our understanding of the epitopes recognized by HLA-specific antibodies. In the first such paper, Zachary and Lefell (Baltimore) provide an insight into the different features of HLA matching, focusing on the decreasing effect of matching influence over recent decades. A more sophisticated approach to matching is now employed, which does not treat all mismatches as equal, but rather considers epitope mismatches in the context of the patients’ HLA immunological profile including desensitization procedures. When HLA mismatches present in a first donor and are repeated in a patient having a second transplant (repeat mismatches), it appears that they are only associated with increased risk of graft loss in patients who are HLA sensitized or those recipients who underwent nephrectomy of the first failed graft. It appears that reexposure to HLA class 2 is more damaging than class 1.

Rene Duquesnoy (Pittsburgh) summarizes the historical aspects surrounding the development of the epitope and eplet concept of HLA antibody recognition, for which he has been the preeminent pioneer. The original concept of treating each HLA serologically defined antigen as a single entity was overturned by the demonstration using sequence data, that each HLA molecule consists of multiple epitopes, some unique, and some shared with other antigens. The approach that arose from this realization was the use of epitope data in the selection of organ donors, particularly for sensitized patients but also recognizing permissible HLA mismatches in non-sensitized patients. In this excellent review, we are taken on a journey from the initial serological demonstration of antibodies, which broadly recognized HLA antigens, to the study of these antibodies at the molecular level, and the demonstration that there are polymorphic triplets of amino acids which are the signature of the epitope and are referred to as eplets. As this concept gains wider acceptance among transplant units, HLA epitope matching will become the method of choice, replacing the historical method of HLA antigen matching currently in use in many centers.

The final contribution, despite being tangential to the topic under discussion, provides some insight into allograft tolerance in a mouse model. Shen and coworkers (Nanjing, China) utilized a mouse cardiac transplant model to generate T regulatory cells and then transfer them to an intestinal transplant model where they observed prolonged survival. The use of T regulatory cells with co-stimulation blockade appeared to be a successful tolerance model.

In summary, although great progress has been made in the detection of HLA and non-HLA antibodies and their relevance to the outcome of solid organ transplants, there are still challenges ahead. The definition of a hierarchy of alloresponsiveness to HLA epitopes, which is dependent on the HLA molecules expressed by the recipient, may be revealed by the full sequencing of both recipients and donors made possible by next-generation sequencing. Such an approach used in a large collaborative study could make possible immunogenicity grading of epitopes interpreted in the context of individual recipients’ HLA genotypes.

The expansion of antibody screening bead technology to include both HLA and non-HLA epitopes will allow further study of the detrimental or otherwise effect of various classes of antibodies. Although many of the non-HLA antibodies are considered auto in nature, an extensive study of the sequences of these genes in populations may reveal epitopes, which include polymorphic residues thus creating the possibility of using this information to select the most appropriate donor for each recipient.

Finally, many transplant recipients display mixtures of IgG isotypes, both complement and non-complement fixing. The mechanism by which mixtures of these antibodies influence rejection *via* both complement binding mechanisms and by ADCC and how these relate to both acute and chronic antibody rejection requires further study to fully understand the subtleties of these antibody interactions.

We recommend that all those interested in the role of antibodies in their many forms on the outcome and survival of a range of allograft types take advantage of this free online series of papers. They are contributed by leaders in the field of clinical transplantation and represent an up to date summary of the current state of play. Both HLA and non-HLA antibodies are discussed in the context of AMR and the mechanisms, whereby these antibodies compromise the outcome of the graft that are discussed.

The editors are pleased to be able to present this collection of papers to you as a series and feel that it represents an important contribution to the current literature on this topic.

## Author Contributions

All authors listed have made substantial, direct, and intellectual contribution to the work and approved it for publication.

## Conflict of Interest Statement

The authors declare that the research was conducted in the absence of any commercial or financial relationships that could be construed as a potential conflict of interest.

